# Identification of canonical pyroptosis-related genes, associated regulation axis, and related traditional Chinese medicine in spinal cord injury

**DOI:** 10.3389/fnagi.2023.1152297

**Published:** 2023-05-18

**Authors:** Wenshan Shan, Shuang Li, Zongsheng Yin

**Affiliations:** ^1^Department of Orthopedics, The First Affiliated Hospital of Anhui Medical University, Hefei, Anhui, China; ^2^The Key Laboratory of Microbiology and Parasitology of Anhui Province, Anhui Medical University, Hefei, Anhui, China

**Keywords:** spinal cord injury (SCI), neuroinflammation, pyroptosis, transcription factors (TF), miRNA, ceRNA, traditional Chinese medicine (TCM)

## Abstract

Neuroinflammation plays an important role in spinal cord injury (SCI), and pyroptosis is inflammatory-related programmed cell death. Although neuroinflammation induced by pyroptosis has been reported in SCI, there is a lack of systematic research on SCI pyroptosis and its regulation mechanism. The purpose of this study was to systematically analyze the expression of pyroptosis-related genes (PRGs) in different SCI models and associated regulation axis by bioinformatics methods. We downloaded raw counts data of seven high-throughput sequencings and two microarray datasets from the GEO database, classified by species (rat and mouse) and SCI modes (moderate contusive model, aneurysm clip impact-compression model, and hemisection model), including mRNAs, miRNAs, lncRNAs, and circRNAs, basically covering the acute, subacute and chronic stages of SCI. We performed differential analysis by R (DEseq2) or GEO2R and found that the AIM2/NLRC4/NLRP3 inflammasome-related genes, GSDMD, IL1B, and IL18, were highly expressed in SCI. Based on the canonical NLRP3 inflammasome-mediated pyroptosis-related genes (NLRP3/PRGs), we constructed transcription factors (TFs)–NLRP3/PRGs, miRNAs- Nlrp3/PRGs and lncRNAs/circRNAs/mRNAs–miRNA- Nlrp3/PRGs (ceRNA) networks. In addition, we also predicted Traditional Chinese medicine (TCM) and small, drug-like molecules with NLRP3/PRGs as potential targets. Finally, 39 up-regulated TFs were identified, which may regulate at least two of NLRP3/PRGs. A total of 7 down-regulated miRNAs were identified which could regulate Nlrp3/PRGs. ceRNA networks were constructed including 23 lncRNAs, 3 cicrRNAs, 6 mRNAs, and 44 miRNAs. A total of 24 herbs were identified which may with two NLRP3/PRGs as potential targets. It is expected to provide new ideas and therapeutic targets for the treatment of SCI.

## Introduction

Spinal cord injury (SCI) often leads to irreversible sensory and motor dysfunction, resulting in lifelong disability of patients and a heavy burden on society (Khorasanizadeh et al., 2019; Keihanian et al., 2022). Currently, there is no effective cure for SCI. The pathological process of SCI is usually divided into primary injury and secondary injury, and the latter is the main research direction at present (Anjum et al., 2020). Cytokines/chemokines produced by residing microglia, astrocytes, peripherally derived immune cells, and endothelial cells, among which TNF, IL-1, and IL-6 have been extensively studied, are upregulated within hours after initial spinal cord injury, leading to extensive infiltration of immune cells. These cells continue to produce more inflammatory mediators that induce neuroinflammation, leading to nerve cell death and inhibiting axon regeneration and functional recovery after spinal cord injury. Neuroinflammation plays an important role in secondary injury and studying the mechanism of neuroinflammation may provide potential therapeutic targets for treating SCI (DiSabato et al., 2016; Hellenbrand et al., 2021).

Pyroptosis is inflammatory-related programmed cell death and the gasdermin protein family is the executioner of pyroptosis. The most common form is the assembly of NLRP3 inflammasome, which cleaves and activates Casp1. Activated Casp1 on the one hand promotes the maturation of inflammatory factors such as IL-1β and cleaves Gsdmd on the other hand. The Gsdmd-N accumulates and forms pores on the membrane resulting in the release of cell contents. Finally, the cells swell and necrosis, inducing a strong inflammatory response (He et al., 2015; Shi et al., 2015; Sborgi et al., 2016).

Neuroinflammation induced by pyroptosis has been reported in SCI (Mortezaee et al., 2018; Xu et al., 2020; Al Mamun et al., 2021). Exploring the pyroptosis-related genes and their regulatory mechanism in spinal cord injury is beneficial to find potential therapeutic targets for SCI from the perspective of neuroinflammation. Because of the particularity of SCI disease, the human body sample study is fewer, and most of the studies are based on animal models. There are many SCI models and the period is large (acute, subacute, and chronic phases). A single animal model cannot fully reflect the pathological process of the disease in the human body. At present, there is a lack of systematic research on SCI pyroptosis and its regulation mechanism. And the main purpose of this study is to find some common pyroptosis-related genes and explore their regulatory mechanisms by comparing gene expression in SCI models of different species, different modeling methods, and different periods.

**FIGURE 1:**
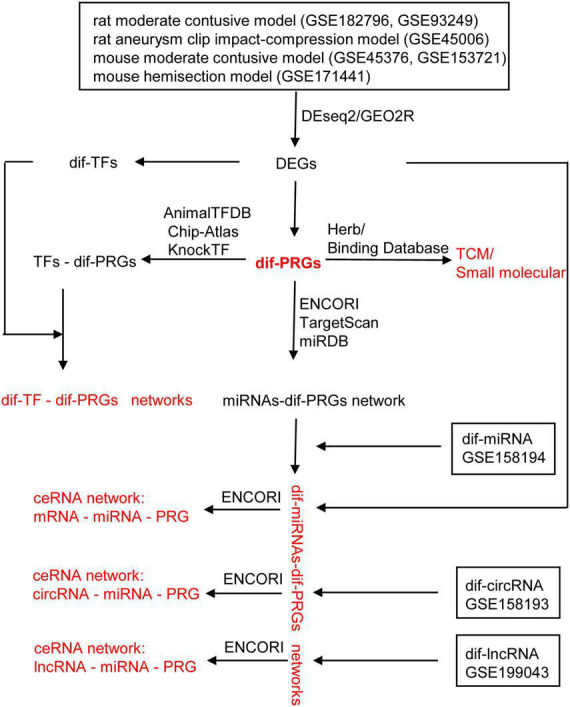
The work flowchart of the study.

In this study, bioinformatics methods were used to find that pyroptosis-related genes (PRGs): AIM2/NLRC4/NLRP3 inflammasome-related genes, GSDMD, IL1B, and IL18 were up-regulated in rat moderate contusive model, rat aneurysm clip impact-compression model, mouse moderate contusive model, and mouse hemisection model, basically covering the acute, subacute and chronic stages of SCI. Based on the canonical NLRP3 inflammasome-mediated pyroptosis-related genes (NLRP3/PRGs), we constructed transcription factors (TFs)–NLRP3/PRGs, miRNAs- Nlrp3/PRGs and lncRNAs/circRNAs/mRNAs–miRNA- Nlrp3/PRGs (ceRNA) networks. In addition, we also predicted Traditional Chinese medicine (TCM) and small, drug-like molecules with NLRP3/PRGs as potential targets. It is expected to provide new ideas and therapeutic targets for treating SCI (Figure 1).

## Materials and methods

### Datasets

We downloaded raw counts data of 7 high-throughput sequencings and two microarray datasets from the GEO database, classified by species and SCI modes, including mRNAs, miRNAs, lncRNAs, and circRNAs. And mRNA datasets basically covered the acute, subacute, and chronic spinal cord injury periods. Information on these datasets is shown in Table 1. To identify differentially expressed mRNA (dif-mRNA), miRNA (dif-miRNA), lncRNA (dif-lncRNA), and circRNA (dif-circRNA), we used the DEseq2 package in R (Love et al., 2014) for high-throughput sequencing datasets and GEO2R^1^ for microarray datasets with | log2(fold-change) | > 0.6 and *P*-value < 0.05.

**TABLE 1:** Basic information of the 9 datasets.

	SCI Model	GEO	Platform	Tissue	Transcriptome	Time points
Mus musculus	Hemisection	GSE171441	GPL16417	Spinal cord	mRNA	3D, 14D, 35D
	Moderate contusive	GSE45376	GPL13112	Spinal cord	mRNA	2D, 7D
		GSE153721	GPL13112	Spinal cord	mRNA	1M, 3M
		GSE158193	GPL21103	Spinal cord	circRNA	3D
		GSE158194	GPL17021	Spinal cord	miRNA	3D
		GSE199043	GPL24247	Spinal cord	lncRNA	7D
Rattus norvegicus	Moderate contusive	GSE182796	GPL23040 Array	Spinal cord	mRNA	7D, 28D
		GSE93249	GPL14844	Spinal cord	mRNA	1M, 3M, 6M
	Aneurysm clip impact-compression	GSE45006	GPL14844 Array	Spinal cord	mRNA	1D, 3D, 1W, 2W, 8W

### Identification of the expression of PRGs at different stages of SCI models constructed in different species and different modeling methods

A total of 33 hsa-PRGs were obtained from previous literature (Lin et al., 2021; Ye et al., 2021) and converted into mmu-PRGs and rno-PRGs by biomaRt package in R (Supplementary Table 1). The differentially expressed PRGS, the absolute value of log2(fold-change) > 0.6 and *P*-value < 0.05, were presented in the form of heat maps drawn by log2TPM + 1 (R package “pheatmap”).

### TFs—NLRP3/PRGs networks construction

The genes associated with canonical NLRP3 inflammasome-mediated pyroptosis include NLRP3, PYCARD, CASP1, GSDMD, IL1B, and IL18, which are all upregulated in SCI. We used the intersection of AnimalTFDB3.0,^2^ Chip-Atlas,^3^ and KnockTF^4^ to predict the upstream TFs of these six genes. To obtain differentially expressed TF in SCI, we first converted dif-TFs in rat and mouse SCI models into human TFs and then intersected these genes. Finally, TFs –NLRP3/PRGs networks were constructed by Cytoscape 3.8 (Shannon et al., 2003).

### Gene ontology (GO) enrichment analysis and the Kyoto encyclopedia of genes (KEGG) pathway analysis

We used DAVID^5^ to perform GO/KEGG analysis on the up-regulated TFs in TFs –NLRP3/PRGs networks and used the ggplot2 package in R to draw dotplot maps. The cutoff criterion for the analysis was *P*-value < 0.05.

### Protein-protein interaction (PPI) network construction and Hub genes identification

We combined six TFs –NLRP3/PRGs networks into one network, reserved only the up-regulated TFs, and each TF regulates at least two NLRP3/PRGs. We used STRING^6^ and Cytoscape 3.8. to construct PPI Network, and hub genes were identified by MCODE.

### The mmu-miRNAs- Nlrp3/PRGs network construction

We used ENCORI,^7^ miRWalk,^8^ TargetScan^9^ and miRDB^10^ to predict the mmu-miRNAs that may directly regulate Nlrp3/PRGs, reserved the intersection of at least two databases. Combined with dif-miRNAs, we constructed the miRNAs- Nlrp3/PRGs network by Cytoscape 3.8.

### The mmu- lncRNAs -miRNA- Nlrp3/PRGs network construction

We selected the dif-miRNA in the miRNAs-Nlrp3/PRGs network or the mmu-miRNAs that could regulate at least two Nlrp3/PRGs and predicted mmu-lncRNAs that could directly bind to these miRNAs by ENCORI. Only upregulated lncRNAs were retained.

### The mmu-circRNAs -miRNA- Nlrp3/PRGs network construction

We selected the dif-miRNA in the miRNAs-Nlrp3/PRGs network or the miRNAs that could regulate at least two NLRP3/PRGs and predicted mmu-circRNAs that could directly bind to these miRNAs by ENCORI. Only upregulated circRNAs were retained.

### The mmu-mRNAs-miRNA- Nlrp3/PRGs network construction

We screened the up-regulated mRNA expression from mouse contusion and mouse hemisection model at each time point and then used ENCORI to predict the mRNAs that could form ceRNA with Nlrp3/PRGs. Only up-regulated mRNAs were retained.

### TCM- NLRP3/PRGs network construction

We used Herb^11^ to predict TCM with NLRP3/PRGs as potential targets and constructed TCM- NLRP3/PRGs networks by Cytoscape 3.8.

### Molecular- NLRP3/PRGs network construction

We used the Binding Database^12^ to predict small, drug-like molecules with NLRP3/PRGs as potential targets and constructed molecular-NLRP3/PRGs networks by Cytoscape 3.8.

### Animal model and quantitative real-time polymerase chain reaction (qRT-PCR)

All protocols were approved by the Animal Ethics Committee of Anhui Medical University. Specific pathogen-free female adult Sprague-Dawley (SD) rats underwent T9-T11 laminectomy, and then a 10 g rod was dropped from a height of 50 mm to the exposed spinal cord to induce T10 contusive spinal cord injury in rats. After surgery, the rats’ bladder was pressed twice a day for urination until the bladder function recovered. One week after surgery, the rats were sacrificed after anesthesia, and the spinal cord tissue in the injured area was obtained. Total RNA from spinal cord tissues was extracted using TRIzol Reagent (Invitrogen, USA). The Evo M-MLV RT Premix for qPCR kit (Accurate Biotechnology, China) was used in mRNA reverse transcription, and the expression of mRNA was analyzed by Q-PCR using SYBR Green Premix Pro Taq HS qPCR Kit (Accurate Biotechnology, China) with β-actin as endogenous controls. The data was obtained using LightCycler 96 (Roche, Swiss), and 2^–ΔΔ*Ct*^ method was used to analyze relative expression levels. Rat ACTB Endogenous Reference Genes Primers (10 μM) were purchased from Sangon Biotech (Shanghai, China), and the rest of the primers were designed and synthesized by Sangon Biotech (Shanghai, China). The primer sequences are shown in Table 2.

**TABLE 2:** Primer sequences.

mRNA	Primer sequences
Foxm1	5′-GCGGACATCCAGAGCATCATCAC -3′ (Foward)
	5′-TGCTGGTTTGGGCTTGAGATTGAG -3′ (Reverse)
Gata1	5′-CGAGGAACCGCAAGGCATCTG -3′ (Foward)
	5′-CACCAGCTACCACCATGAATCCAC -3′ (Reverse)
Myb	5′- CGTCGCAAGGTGGAACAGGAAG-3′ (Foward)
	5′-CTGGCTAGTTGGAGGAGGTGAGG-3′ (Reverse)
Atf3	5′-CCTCTCACCTCCTGGGTCACTG -3′ (Foward)
	5′-TGCTTGTTCTGGATGGCGAATCTC -3′ (Reverse)
Tp53	5′-CCTTACCATCATCACGCTGGAAGAC -3′(Foward)
	5′-AGGACAGGCACAAACACGAACC -3′ (Reverse)
Nfya	5′- AGACAGGAGCCAATACCAACACAAC-3′ (Foward)
	5′- GGGATTCTTTGGATAGCAGGCACAG-3′ (Reverse)
Elk3	5′-CCTGTACTCGTCCTTCACCATCAAC -3′ (Foward)
	5′-GGCTGTCATCACATGGCTCCTC -3′ (Reverse)
Maf	5′-ACGTCCTGGAGTCGGAGAAGAAC -3′ (Foward)
	5′- GAACCCGCTGCTCACCAACTTC-3′ (Reverse)

### Statistical analysis

**FIGURE 2:**
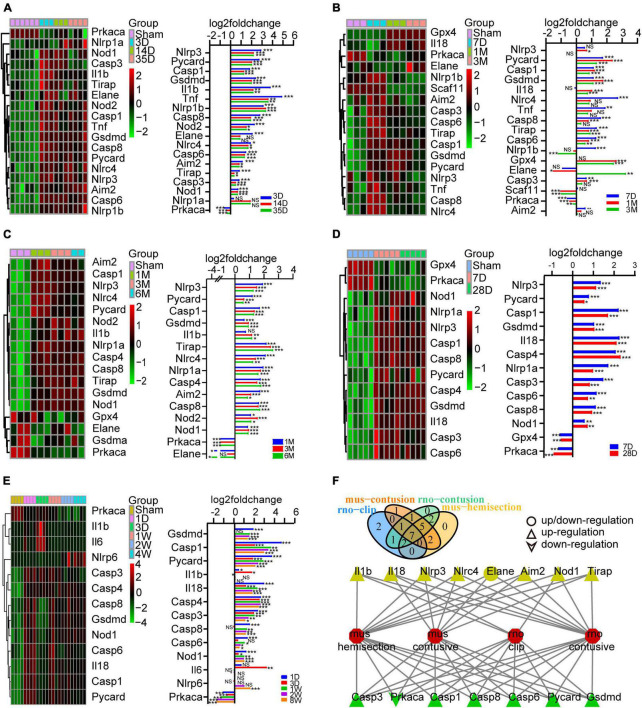
Identification of the expression of PRGs at different stages of SCI models constructed in different species and modeling methods. **(A)** Heatmap (left) of the PRGS and histogram of their log2foldchange (right) between the sham and 3D, 14D, 35D after SCI in mouse hemisection model. **(B)** Heatmap (left) of the PRGS and histogram of their log2foldchange (right) between the sham and 7D, 1M, 3M after SCI in mouse moderate contusive model. **(C,D)** Heatmap (left) of the PRGS and histogram of their log2foldchange (right) between the sham and 7D, 28D, 1M, 3M, 6M after SCI in rat contusive model. **(E)** Heatmap (left) of the PRGS and histogram of their log2foldchange (right) between the sham and 1D, 3D, 1W, 2W, 4W after SCI in rat aneurysm clip impact-compression model. **(F)** The intersection of the differentially expressed PRGs of the four (green) or three (yellow) SCI models. *P < 0.05, **P < 0.01, ***P < 0.001.

The differential expression of PRG between the SCI group (at each time point) and the Sham group was expressed as log2foldchage. The *p*-value was obtained using the R package “DEseq2” or GEO2R (Figure 2). GO/KEGG analysis and the *p*-value were performed by DAVID (Figure 5). The data of qRT-PCR were expressed as mean ± SEM, GraphPad Prism software was used to perform statistical analyses and the significance of differences between the two groups was using an unpaired 2-tailed *t*-test (Figure 7). *P* < 0.05 was considered to indicate significant differences.

## Results

### Identification of the expression of PRGs at different stages of SCI models constructed in different species and different modeling methods

To explore the expression of PRGs in SCI, we downloaded 6 SCI datasets from the GEO database, including rat moderate contusive model (GSE182796, GSE93249), rat aneurysm clip impact-compression model (GSE45006), mouse moderate contusive model (GSE45376, GSE153721) and mouse hemisection model (GSE171441), basically covering the acute, subacute and chronic stages of SCI. A total of 17 PRGs were upregulated and 1 downregulated in mouse hemisection model (Figure 2A). A total of 13 PRGs were upregulated, 2 downregulated, and 2 up/downregulated in mouse moderate contusive model (Figure 2B). A total of 16 PRGs were upregulated and 3 downregulated in rat moderate contusive model (Figures 2C, D). A total of 12 PRGs were upregulated and 1 downregulated in rat aneurysm clip impact-compression model (Figure 2E). We then took the intersection of the differentially expressed PRGs of the four SCI models. Finally, six genes (Casp1, Casp3, Casp6, Casp8, Gsdmd, Pycard) were up-regulated and 1 gene (Prkaca) was down-regulated in four SCI models seven genes (Aim2, Nlrc4, Nlrp3, Tirap, Il18, Nod1, Il1b) were up-regulated, and 1 gene (Elane) was up/downregulated in three models (Figure 2F). We selected the canonical NLRP3 inflammasome-mediated pyroptosis-related genes (Nlrp3, Pycard, Casp1, Gsdmd, Il1b, and Il18) for subsequent analysis.

### TFs—NLRP3/PRGs networks construction and GO/KEGG analysis of up-regulated TFs

**FIGURE 3:**
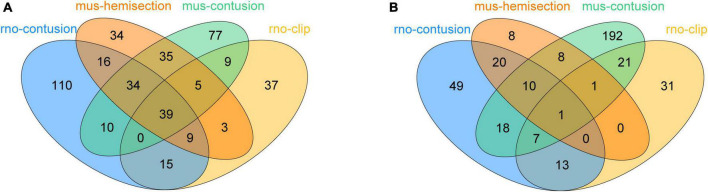
Dif-TFs in four SCI models: up-regulated **(A)** and down-regulated **(B)**.

**FIGURE 4:**
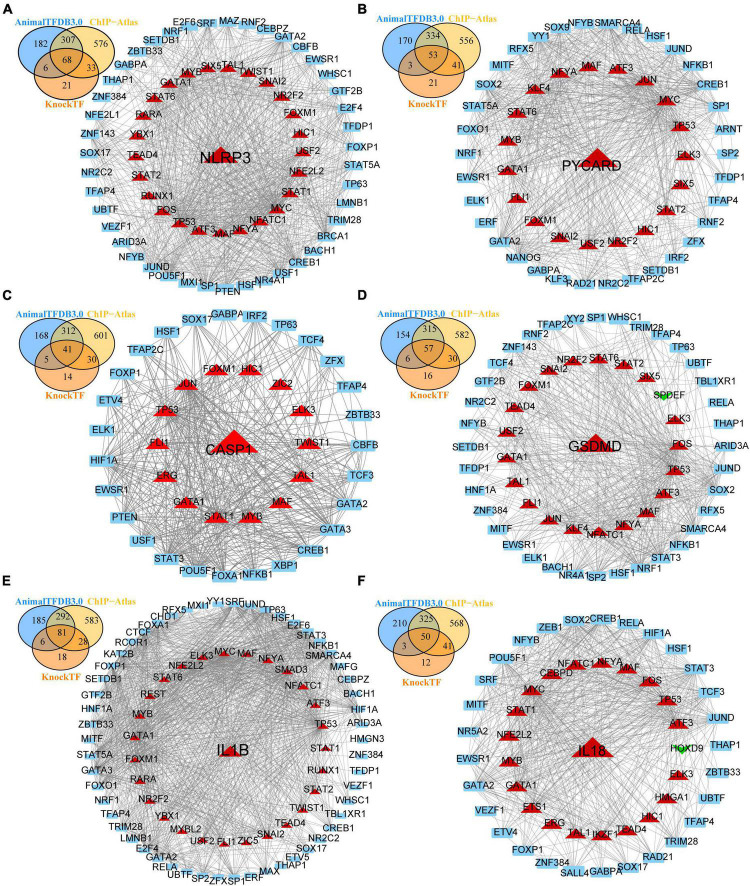
Transcription factors (TFs)—NLRP3/PRGs networks construction. **(A)** TFs-NLRP3 networks, **(B)** TFs-PYCARD networks, **(C)** TFs-CASP1 networks, **(D)** TFs-GSDMD networks, **(E)** TFs-IL1B networks, **(F)** TFs-IL18 networks. Red: up-regulated, green: down-regulated.

**FIGURE 5:**
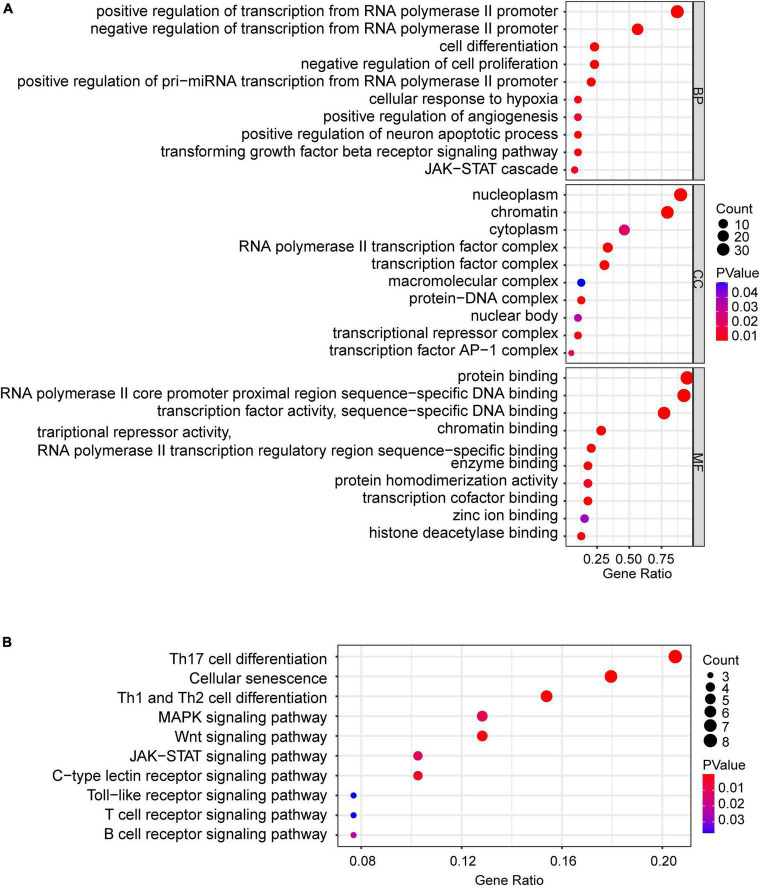
Bubble maps of GO and KEGG pathway analysis of up-regulated TFs (a total of 39) in TFs—NLRP3/PRGs networks. **(A)** 10 of each selected GO-BP, GO-MF, and GO-CC terms enriched, **(B)** 10 selected pathways enriched. *P* < 0.05.

To obtain differentially expressed TFs in SCI, we first converted dif-TFs in rat and mouse SCI models into human TFs and then retained TFs that express differences in at least two models, Finally, we obtained 175 up-regulated (Figure 3A) and 99 down-regulated TFs (Figure 3B and Supplementary Table 2). We then used the intersection of AnimalTFDB3.0, Chip-Atlas, and KnockTF to predict the upstream transcription factors of six NLRP3/PRGs (NLRP3, PYCARD, CASP1, GSDMD, IL1B, and IL18). Finally, 68 TFs-NLRP3 were obtained, and 25 of them were up-regulated (Figure 4A); 53 TFs-PYCARD were obtained, and 19 of them were up-regulated (Figure 4B); 41 TFs-CASP1 were obtained, and 14 of them were up-regulated (Figure 4C); 57 TFs-GSDMD were obtained, and 20 of them were up-regulated and 1 was down-regulated (Figure 4D); 81 TFs-IL1B were obtained, and 27 of them were up-regulated (Figure 4E); 50 TFs-IL18 were obtained, and 20 of them were up-regulated and 1 was down-regulated (Figure 4F). We then performed GO Enrichment Analysis (Figure 5A) and KEGG Pathway Analysis (Figure 5B) on up-regulated TFs in TFs—NLRP3/PRGs networks (a total of 39) and found they were significantly associated with MAPK signaling pathway, Wnt signaling pathway, JAK-STAT signaling pathway and so on.

### Protein-protein interaction (PPI) network and Hub genes identification

**FIGURE 6:**
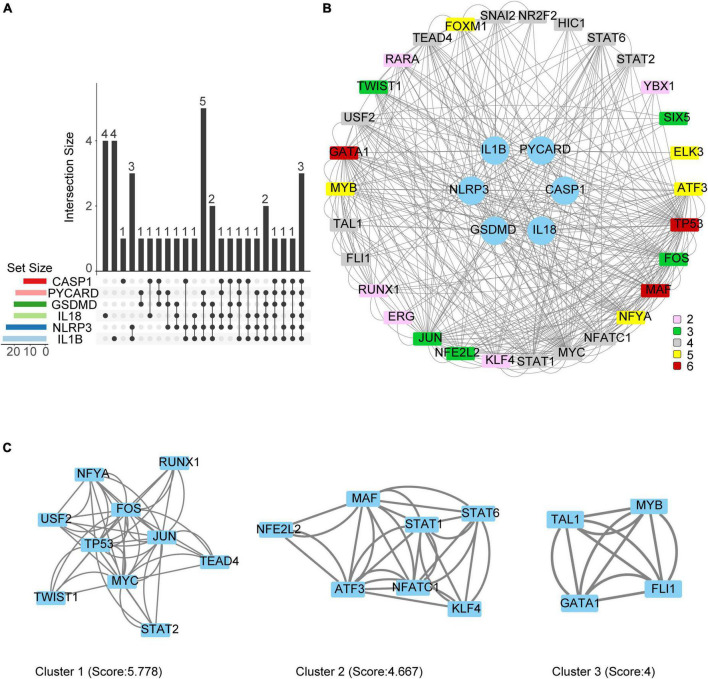
Protein-Protein interaction (PPI) network and Hub genes identification. **(A)** Vennpie of up-regulated TFs in TFs—NLRP3/PRGs networks. **(B)** PPI network of up-regulated TFs in TFs—NLRP3/PRGs networks. Different colors represent the number of PRGs regulated by TF. **(C)** Three sub-network modules were identified by MCODE.

**FIGURE 7:**
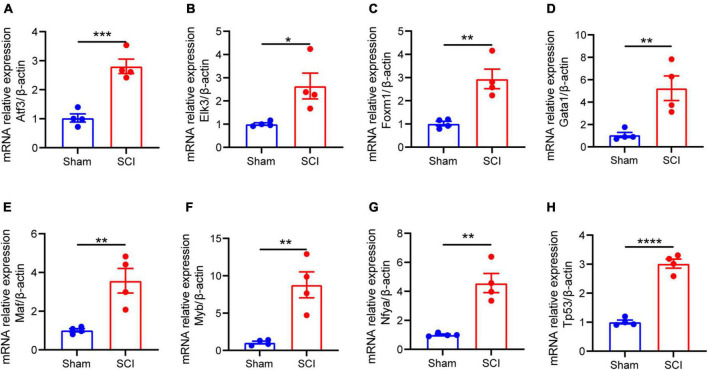
The mRNA expression of 8 TFs (regulate at least five of NLRP3/PRGs) in rat moderate contusive model. **(A–H)** The expression of TFs in sham and SCI groups was detected by qRT–PCR (*n* = 4 per group). Data are represented as mean ± SEM. *P < 0.05, **P < 0.01, ***P < 0.001, ****P < 0.0001.

We reserved only the up-regulated TFs in TFs—NLRP3/PRGs networks and each TF regulates at least two of NLRP3/PRGs (Supplementary Table 3). Finally, we obtained 3 TFs (MAF, TP53, GATA1) regulate six of NLRP3/PRGs, 5 TFs (MYB, FOXM1, ELK3, ATF3, NFYA) regulate five of NLRP3/PRGs, 12 TFs (SNAI2, NR2F2, HIC1, STAT6, STAT2, NFATC1, MYC, STAT1, FLI1, TAL1, USF2, TEAD4) regulate four of NLRP3/PRGs, 5 TFs (JUN, NFE2L2, FOS, SIX5, TWIST1) regulate three of NLRP3/PRGs, 5 TFs (RARA, YBX1, KLF4, ERG, RUNX1) regulate two of NLRP3/PRGs. STRING and Cytoscape 3.8. were used to construct PPI Network (Figures 6A, B). We also detected the mRNA expression of 8 TFs (regulate at least five of NLRP3/PRGs) in rat moderate contusive models to verify their upregulation in spinal cord injury (Figures 7A–H). In addition, three sub-network modules were aggregated and extracted from the PPI network using the Cytoscape plug-in MCODE (Figure 6C).

### The mmu-miRNAs- Nlrp3/PRGs network construction

**FIGURE 8:**
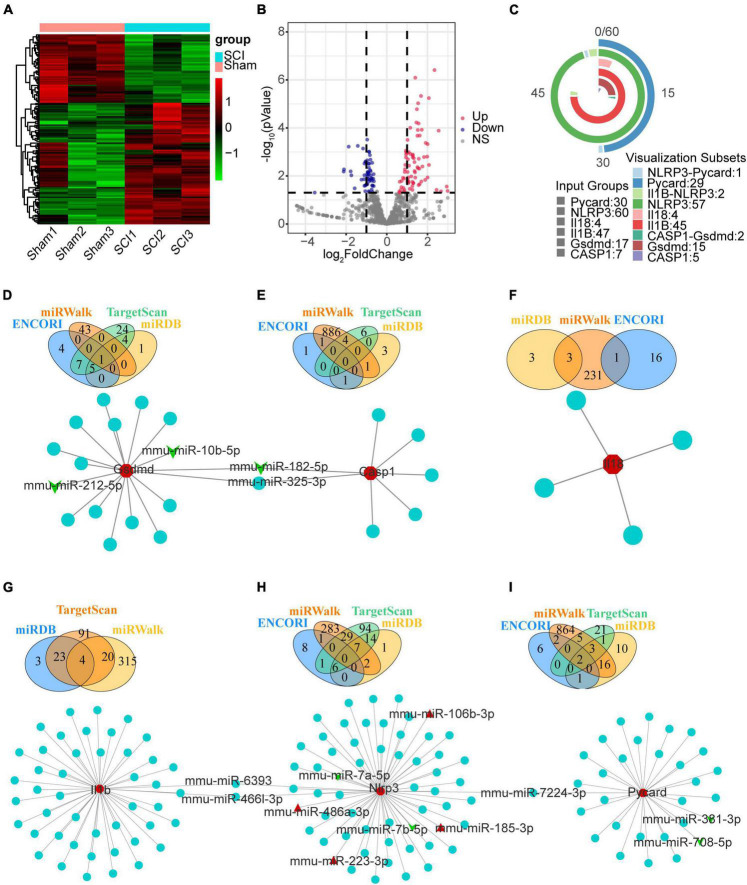
The mmu-miRNAs- Nlrp3/PRGs network construction. **(A)** Heatmap (green: low expression level; red: high expression level) of the dif-miRNA, **(B)** volcano plot for miRNAs between the sham and SCI group. | logFC| > 0.6 and *p* < 0.05 **(C)** vennpie of miRNAs in miRNAs- Nlrp3/PRGs network. **(D–I)** miRNAs- Nlrp3/PRGs networks: miRNAs-Nlrp3 **(H)**, miRNAs-Pycard **(I)**, miRNAs-Casp1 **(E)**, miRNAs-Gsdmd **(D)**, miRNAs-Il1b **(G)**, miRNAs-Il18 **(F)**. Red: up-regulated, green: down-regulated.

We downloaded raw counts data of GSE158194 from the GEO database and identified differentially expressed miRNA by the DEseq2 package in R. Finally, 101 up-regulated, and 57 down-regulated miRNA were obtained (Figures 8A, B and Supplementary Table 3). We then used ENCORI, miRWalk, TargetScan, and miRDB to predict the mmu-miRNAs that may directly regulate Nlrp3/PRGs (Nlrp3, Pycard, Casp1, Gsdmd, Il1b, Il18), reserved the intersection of at least two databases. Finally, 60 miRNAs-Nlrp3 were obtained, and 4 of them were up-regulated and two down-regulated (Figure 8H); 30 miRNAs-Pycard were obtained, and 2 of them were down-regulated (Figure 8I); 7 miRNAs-Casp1 were obtained, and 1 of them were down-regulated (Figure 8E); 17 miRNAs-Gsdmd were obtained, and 3 of them were down-regulated (Figure 8D); 47 miRNAs-Il1b were obtained (Figure 8G); 4 miRNAs-Il18 were obtained (Figure 8F). We take the intersection of six miRNAs- Nlrp3/PRGs networks (Figure 8C) and got five miRNAs that could regulate at least two of Nlrp3/PRGs: miR-182-5p/miR-325-3p—Gsdmd/Casp1, miR-6393/miR-466I-3p—Nlrp3/Il1b, miR-7224-3p—Nlrp3/Pycard.

### The mmu-ceRNA network construction

**FIGURE 9:**
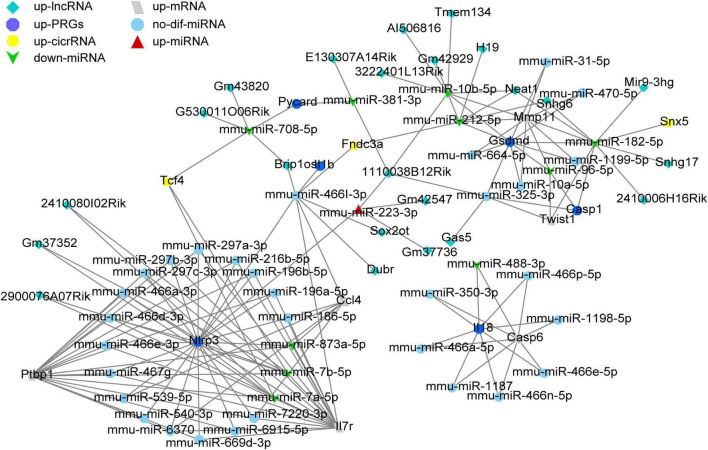
The ceRNA network construction. A total of 23 lncRNAs, 3 cicrRNAs, 6 mRNAs, and 44 miRNAs constructed lncRNA/cicrRNA/mRNA—miRNA—Nlrp3/ PRGs ceRNA networks.

To construct ceRNA networks (lncRNA/cicrRNA/mRNA—miRNA—Nlrp3/PRGs), we selected the dif-miRNA in the miRNAs-Nlrp3/PRGs network or the miRNAs that could regulate at least two Nlrp3/PRGs (mmu-miR-106b-3p, mmu-miR-10b-5p, mmu-miR-182-5p, mmu-miR-185-3p, mmu-miR-212-5p, mmu-miR-223-3p, mmu-miR-325-3p, mmu-miR-381-3p, mmu-miR-466l-3p, mmu-miR-486b-3p, mmu-miR-6393, mmu-miR-708-5p, mmu-miR-7224-3p, mmu-miR-7a-5p, mmu-miR-7b-5p) and predicted mmu-circRNAs and mmu-lncRNAs that could directly bind to these miRNAs by ENCORI. Only upregulated circRNAs and lnc RNA were retained. We screened the up-regulated mRNAs expression from mouse contusion and mouse hemisection model at each time point and then used ENCORI to predict the mRNAs that could form ceRNA with Nlrp3/PRGs. Only up-regulated mRNAs were retained. Finally, we obtained 23 lncRNAs, 3 cicrRNAs, 6 mRNAs, and 44 miRNAs, and constructed ceRNA networks by Cytoscap 3.8 (Figure 9).

### TCM/small molecular- NLRP3/PRGs network construction

**FIGURE 10:**
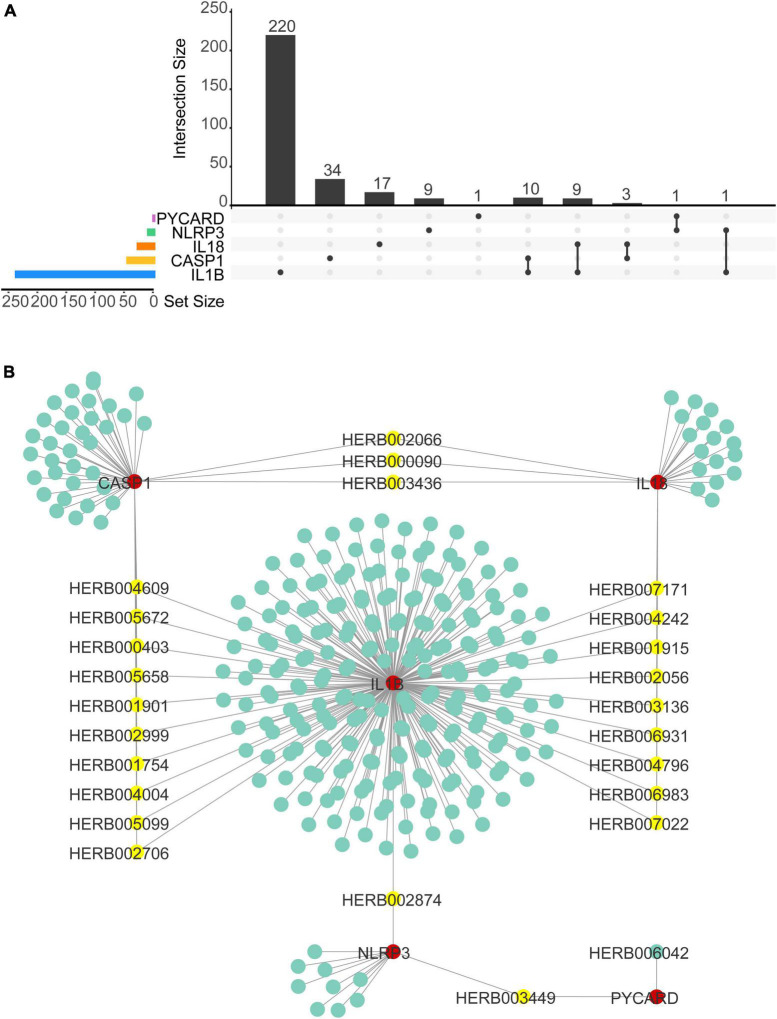
Traditional Chinese medicine (TCM)–NLRP3/PRGs network construction. **(A)** Upset plot of TCMs of TCMs–NLRP3/PRGs network. **(B)** TCMs–NLRP3/ PRGs network.

We used Herb Database to predict TCM with NLRP3/PRGs (NLRP3, PYCARD, CASP1, IL1B, IL18) as potential targets. We obtained 11 TCM-NLRP3, 2 TCM-PYCARD, 47 TCM-CASP1, 234 TCM-IL1B, 29 TCM-IL18. By taking the intersection of these five networks, we get three herbs that target both NLRP3 and IL18, 10 herbs that target both CASP1 and IL1B, 9 herbs that target both IL18 and IL1B, 1 herb that targets both NLRP3 and IL1B, 1 herb that targets both NLRP3 and PYCARD. We then constructed TCM- NLRP3/PRGs networks by Cytoscap 3.8 (Figures 10A, B). We also used the Binding Database to predict small, drug-like molecules with NLRP3/PRGs as potential targets. We obtained 162 molecule-NLRP3 (Supplementary Figure 1), 560 molecular-ASC (Supplementary Figure 2), 931 molecular-CASP1 (Supplementary Figure 3), 188 molecular–IL1B (Supplementary Figure 4) and constructed molecular-NLRP3/PRGs networks by Cytoscap 3.8. By taking the intersection of these five networks, we obtained 1 small molecular Ac-Yvad-cho (PubChem CID 5311139) that target both CASP1 and IL1B (Supplementary Figure 5).

## Discussion

**FIGURE 11:**
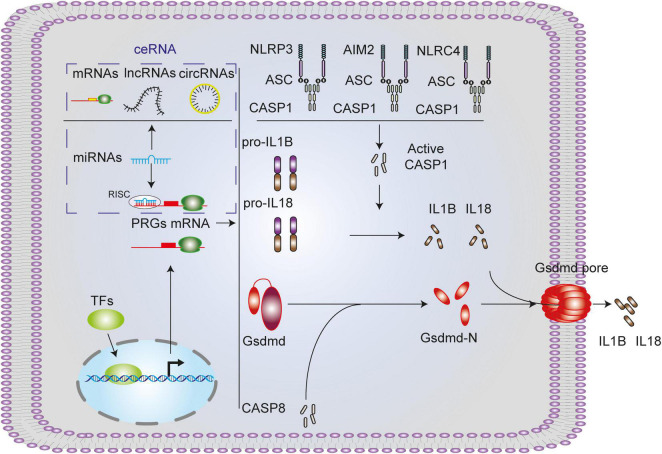
Canonical pyroptosis-related genes and associated regulation axis in spinal cord injury. NLRP3/AIM2/NLRC4 inflammasome activates CASP1, activated CASP1 or CASP8 cleaves GSDMD, GSDMD-N forms pores on the cell membrane, and mature IL1B and IL18 promoted by CASP1 extracellular released into extra cell from the pores.

The gasdermin protein family, the executioner of pyroptosis, includes GSDMA, GSDMB, GSDMC, GSDMD, and GSDME (Galluzzi et al., 2018). In this study, Gsdmd is upregulated in four SCI models, which might be the main executor of SCI pyroptosis (He et al., 2015; Liu et al., 2016; Sborgi et al., 2016). We also performed further analysis of the other executioners. Among them, it is well acknowledged that GSDMB is not expressed in rodents. And in all data sets, there is no GSDME-related data. GSDMA and GSDMC were underexpressed or not expressed in the rodents’ SCI models (Supplementary Figures 6A–E). It has been reported that CASP1, CASP4/5/11, and CASP8 can cleave GSDMD, in this study, CASP1 and CASP8 are upregulated in four SCI models and may be involved in GSDMD cleaved in SCI pyroptosis (Kayagaki et al., 2011, 2015; Aglietti et al., 2016; Sarhan et al., 2018). Activation of CASP1 requires the involvement of upstream inflammasome, including NLRP1 inflammasome, NLRP3 inflammasome, AIM2 inflammasome and NLRC4 inflammasome (Poyet et al., 2001; Agostini et al., 2004; Fernandes-Alnemri et al., 2009; Hornung et al., 2009; Liu et al., 2016; Chui et al., 2019). In this study, NLRP3, AIM2, NLRC4, and PYCARD are upregulated in three SCI models and may be involved in CASP1 activation in SCI pyroptosis (Figure 11). In this study, we selected the canonical NLRP3 inflammasome-mediated pyroptosis related genes (NLRP3, PYCARD, CASP1, GSDMD, IL1B, and IL18) for subsequent analysis.

The upregulation of TFs may lead to the upregulation of their target genes. Therefore, we screened the up-regulated TFs in SCI which could regulate NLRP3/PRGs, to find potential targets for inhibiting SCI pyroptosis. A total of 39 TFs were screened out, 16 of which have been reported to be involved in regulating pyroptosis: ERG (Yao et al., 2022), FLI1 (Li et al., 2018, 2019), FOXM1 (Xu et al., 2021), HIC1 (Gao et al., 2021), JUN (Wang et al., 2020), KLF4 (Xiong et al., 2021), USF2 (Sun et al., 2022), STAT2 (Wang et al., 2022), FOS (Wang et al., 2021), NFATC1 (Kai et al., 2020), MYC (Gaikwad et al., 2020), NFE2L2 (Ling et al., 2021), SMAD3 (Zhu et al., 2020), ETS1 (Juan et al., 2022), IKZF1 (Kadono et al., 2022), and HMGA1 (Liang et al., 2022). In addition, we performed KEGG Pathway Analysis on these 39 TFs, which were enriched into multiple signaling pathways, such as MAPK signaling pathway, Wnt signaling pathway, JAK-STAT signaling pathway, and Toll-like receptor signaling pathway.

The miRNAs are a group of non-coding RNAs encoded by the genome with a length of about 20–23 nucleotides. They degrade the mRNA or block its translation by pairing it with the target gene mRNA (Cai et al., 2009; Lu and Rothenberg, 2018). Therefore, we screened the miRNAs in SCI which could regulate NLRP3/PRGs, to find potential targets for inhibiting SCI pyroptosis. In the predicted miRNAs- Nlrp3/PRGs networks, seven (miR-212-5p, miR-10b-5p, miR-182-5p, miR-7a-5p, miR-7b-5p, miR-381-3p, miR708-5p) were down-regulated, which may be involved in the SCI pyroptosis and it has been reported that miR-182-5p can regulate pyroptosis by targeting Gsdmd (Yue et al., 2022).

It’s well known that miRNA can lead to gene silencing by binding mRNA, while ceRNA can regulate gene expression by competitively binding the same miRNA (Kartha and Subramanian, 2014; Tay et al., 2014). At present, ceRNA has been reported to be involved in the regulation of SCI (Kartha and Subramanian, 2014; Tay et al., 2014; Gu et al., 2021). In this study, we constructed lncRNA/cicrRNA/mRNA–miRNA–NLRP3/PRGs ceRNA networks to find potential targets for inhibiting SCI pyroptosis. Finally, we obtained 33 lncRNAs, 6 cicrRNAs, and 6 mRNAs.

The application of TCM and small, drug-like molecules in treating SCI has been widely studied (Dai et al., 2019; Ding et al., 2021; Zhao et al., 2022). The treatment of TCM targeting pyroptosis has been reported in many diseases, but it is seldom used in spinal cord injury (Li et al., 2022; Yan et al., 2022; Zhan et al., 2022). In this study, we predicted Traditional Chinese medicine (TCM) and small, drug-like molecules with NLRP3/PRGs as potential targets. Finally, we obtained 24 herbs and 1 small molecule with two NLRP3/PRGs as potential targets. Among them, Ac-Yvad-cho has been reported to inhibit pyroptosis (Dai et al., 2019; Ding et al., 2021; Zhao et al., 2022).

## Conclusion

We used bioinformatics methods to find that pyroptosis-related genes (PRGs) were upregulated in four SCI animal models. Based on the canonical NLRP3 inflammasome-mediated pyroptosis-related genes (NLRP3/PRGs), We constructed transcription factors (TFs)–NLRP3/PRGs, miRNAs- Nlrp3/PRGs and lncRNAs/cicrRNAs/mRNAs–miRNA- Nlrp3/PRGs (ceRNA) networks. In addition, we also predicted Traditional Chinese medicine (TCM) and small, drug-like molecules with NLRP3/PRGs as potential targets. It is expected to provide new ideas and therapeutic targets for treating SCI.

## Data availability statement

The datasets presented in this study can be found in online repositories. The names of the repository/repositories and accession number(s) can be found in the article/Supplementary material

## Ethics statement

This animal study was reviewed and approved by the Ethics Committee of Anhui Medical University of China.

## Author contributions

ZY designed the study, reviewed, and edited the manuscript. WS drafted the manuscript. WS and SL performed the bioinformatic analysis. All authors contributed to the article and approved the submitted version.

## Footnotes

1
https://www.ncbi.nlm.nih.gov/geo/geo2r/

2
http://bioinfo.life.hust.edu.cn/AnimalTFDB/#!/tfbs_predict

3
http://chip-atlas.org/enrichment_analysis

4
http://www.licpathway.net/KnockTF/browse.php

5
https://david.ncifcrf.gov/

6
https://cn.string-db.org/

7
https://starbase.sysu.edu.cn/agoClipRNA.php?source=mRNA

8
http://mirwalk.umm.uni-heidelberg.de/

9
https://www.targetscan.org/vert_72/

10
http://mirdb.org/index.html

11
http://herb.ac.cn/Search/

12
https://www.bindingdb.org/bind/info.jsp
