# Supplementary figures and images for: Identification of canonical pyroptosis-related genes, associated regulation axis, and related traditional Chinese medicine in spinal cord injury
Source: Front Aging Neurosci. 2023 May 18;15:1152297. doi: 10.3389/fnagi.2023.1152297 (PMC10232751; doi:10.3389/fnagi.2023.1152297)

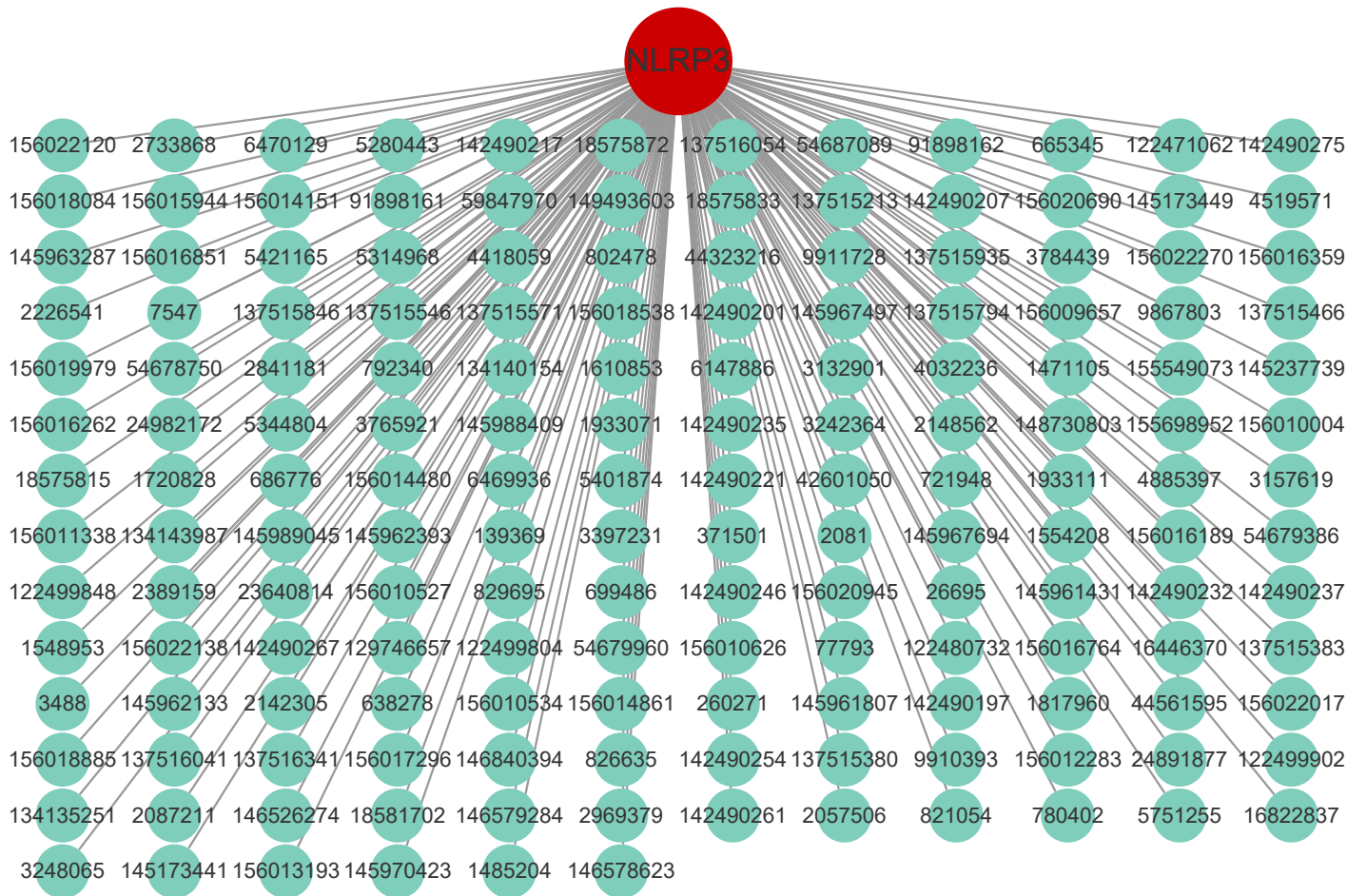

Supplement: Supplementary file 1 [file Data_Sheet_1.PDF]

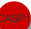

4438998 7189993 4415835 4474690 7152095 1063134 4439654 1177862 4431012 4433178 5201897 7021894 4435540 4071460 4434482 4204435 4064490 4316167 4430344 4426436 4430857 4464640 4027891

4447698 4470795 4442852 4453167 4458504 4440710 4431288 4464306 11872334 4440703 118723238 2249780 2364597 4065079 5826057 1062672 2157459 1674587 2345983 2858592 17201 4427641

5000709 4444847 4437444 1674916 4436003 2470723 4440235 4431000 4430005 9300773 4430078 4430078 12085110 34479338 2707305 4420465 4379721 2188187 4425294 4415702 4464344 44416321 12605009 44218322

4458935 2484347 4441705 4461825 4458983 4464326 44520614 1434150 4444285 45379629 4984646 44341820 1764908 5461849 42518293 4151808 47157039 4427968 4433205 24515452 4201672 4204870 4458947

5052324 1801945 4462343 1582020 11407119 4438557 118723318 1057455 1885848 4440357 4430978 10000909 9810472 4415520 4438930 4427860 2382194 4448263 4466510 4442122 7171895 1740479 4064337

1780 7471100 4433205 4401676 4434660 4443434 4432005 4433195 4417914 4433170 47 2465064 4432109 5463924 2896735 44276614 4440589 4314988 4431863 71883061 4434170 24645309 4430170

4454844 4697375 1741700 7452207 4468404 4440481 4431571 4415825 4634548 4436703 4780625 4433182 12085110 34479338 2707305 4420465 4379721 2188187 4425294 4415702 4464344 44416321 12605009 44218322

12000174 4484700 4386430 4423826 4436824 71882875 4441826 5463843 1032660 8954261 4447042 23645238 1309280 4434818 1024851 1065620 4431843 4427967 442673 4452092 2464520 23645238 4086504

2517850 2345983 1153330 51890 1413344 5823074 493358 1674918 1534595 939554 4415835 4427865 885838 2364523 1151550 4428454 4441620 269844 4427860 11884423 5327305 4431966 267181

44414573 2110122 7163103 518032 4066305 1074916 4521702 4440565 1674069 4427623 11185677 4448450 4430710 4427619 4427844 4433212 526204 2557703 1054504 4425820 105120 4442852 6789036

12702896 4414430 4427845 5488384 11872331 1089833 15888270 4441457 1163649 4854529 2120707 4436503 4433182 3826 2364521 4537973 4315634 721948 4431878 1069300 106770 4086210 4442811

4458940 1488030 4440780 4432934 4438835 4428204 5746324 2464560 11614728 3885 2364524 44314079 4407860 1099954 25217023 1512109 4431878 13144874 4430308 126816 4427984 72331

445880 4440570 4442400 5308163 4427681 4088440 4431878 980805 982805 13413890 4384182 134151945 4468435 916278 4440574 2178949 4427968 4430293 4441570 443180 650642 4441861

4450204 1167854 4415385 5016270 1187804 58822 496437 1581877 4420738 1344607 4434565 228185 44216247 418824 1518187 388528 2364525 443260 4431480 4430695 2364538

44476405 5407701 501731 167126 408652 468787 4420002 4084843 4430515 4433185 1664830 4446437 44414445 4426436 4438900 44345901 4084843 4433231 388884 1268130 4441845 1651213 44405828

246744 4507820 4433205 168710 247585 4440562 4431566 7582194 583842 7583873 4428585 4477016 44276518 4324 4438803 382226 4440705 1588117 3219193 158432 2 1085 939595 4423704

9460485 1168480 1162400 9460484 4415836 7246172 4440716 4438929 44325973 153270 23645235 4073863 44272479 4441069 9514167 4440562 4427963 4438933 4440716 4084443 1074629 4431878 1854267

4441424 4458302 4477975 4433205 443186 171129 442354 4431652 4428006 4433186 4440570 4433170 925156 1187233 170045 443792 4384469 726057 4440570 4432119 2464519 44416321 4427876

10112604 2121777 4441482 4440565 4427637 4281820 4441400 4433206 4430684 4041674 7133367 4441482 4431589 444285 4431877 4439307 4086529 2364524 4437903 4411604 4440589 1674026 4441458

967170 448820 23684 41624 24684 9180370 448024 4438317 4485104 2517854 4 527310 4477861 7345450 474545 406923 12085 4433203 443397 4423307 105947 447827 4432109 4064335

527335 4064335 9345919 1183338 1003119 2381803 4681987 5320701 1527304 4486502 7024549 4427860 8821678 4427840 4432587 14134574 4415705 4440571 4427865 44114826 4440589 44407823 25779140

4445481 580232 444056 443167 44370 4441634 4441584 7182507 4428516 4440710 4 344213 821814 394626 442826 4439519 442525 3330 4440589 4064335 4431510 1181922 4431842 1167820

4431915 4507973 5467845 443585 2464367 4423304 4440707 4063326 4427948 44577362 2364525 44314515 4431908 152599 2 1654277 442767 2387874 4081205 2577932 10011623 4441030 1444481

4458279 2364538 443064 4430709 443334 7182507 443705 442786 158277 2716 4438919 4427865 4441445 445795 4447475 54842 4440562 534867 443484 443844 443487 4440580 538791

4459479 1588282 4438984 4430685 5287858 58382484 10306 4442144 4424145 4434715 2364524 4068548 1871463 3194 7183832 443205 4438802 4440567 4084392 4428202 4438930 1067700 11691540

4464843 1588282 4438984 4430685 444244 443484 4441422 4421458 4434715 2364524 443195 442767 443195 442767 443195 442767 443195 442767 443195 442767 443195 442767 443195

959307 921888 406440 56339 7285105 4427973 5327302 4433187 717385 4884369 25571029 9183338 4049497 1588438 4434186 4440584 11872329 1189109 13725293 4064303 1643907 11872329 416390

11723316 442786 442786 442786 21718 944548 44305 5481838 442786 118783 4440570 4437930 1100298 442828 174444 443484 44305 97840 44305 442786 443305 443305 4441620 146843

4064861 2364524 421820 443170 5468062 65660 1181968 1854267 4431987 4440562 4441621 987540 4428406 1151058 11723303 44315815 70900 4426485 2377933 2364524 4031803 4441845

11524304 2364525 442786 442786 1702319 44305 443484 4441422 4421458 4434715 2364524 2464977 9183338 442845 443195 442786 443195 442786 443195 442786 443195 442786 443195

5458303 44158154 7171624 5000070 4421873 4458303 2364561 4440567 4486259 2288166 4065109 4440570 44865106 4431864 4440567 4482848 4442836 4430303 4064319 4441805 218721 4440570 1169542

7158310 443070 443186 910327 1068419 1180223 443187 4438937 2364522 5487319 7345923 94345 4404857 4427862 4440589 72520 443186 4441442 443407 2364505 443186 443186 4441620 146843

960708 8842580 4435310 2364240 44342718 9821654 4432093 11684819 4463842 4440570 4431909 4068561 4068560 9189184 1070710 4448182 1051889 44315815 4427124 4428266 4415838 1631067 4440581 4434803

4420036 2364525 442786 442786 106324 208328 442786 442786 527370 7183378 4440783 4440783 4440783 442786 442786 527370 442786 442786 442786 718338 4431875 2801815

4064341 443484 11738262 1010330 4438936 930676 2364527 4441452 2364521 2364524 40844421 4427866 11603301 4427968 4438837 2365102 441738 4440571 4458436 4420167 4064338 1354278 4427965

1152394 4432026 18740182 4441822 2364538 1182802 4440782 4441444 4064340 4441431 4440380 4458430 4441678 4084240 4427873 4427869 4424719 4440526 2461838 14804 4431864 2364520 4441675

4434173 406506 1175068 910330 11311 1141746 442784 1002387 4441620 4427780 44216278 4427843 276544 4438931 4427861 332 4421835 442786 442786 237849 1677446 4431873 4064345

4434844 547936 1175068 442784 4438931 4441604 1270186 908262 4440389 4518059 4442834 4440571 4433213 13418439 1083564 4441454 7183830 82 4440567 4431864 4441448

4441482 4440567 2364529 4065104 4433187 4431879 1083510 4434170 23645975 11872330 4064344 900385 2577914 4421872 1354345 4431833 44684367 4058 4425844 4440712 4425874 4064367 527793

44341819 4440712 4438938 4464332 7188314 4431994 2364524 7188304 4441843 888889 4461117 23645971 2364527 1161880 4432128 4442847 9063304 44489205 7171897 2364576 4434170 4441987 12702875

44518288 44414105 4448921 4440028 9694921 684198 4442852 548 4448938 44415814 4427863

Supplement: Supplementary file 3 [file Data_Sheet_3.PDF]

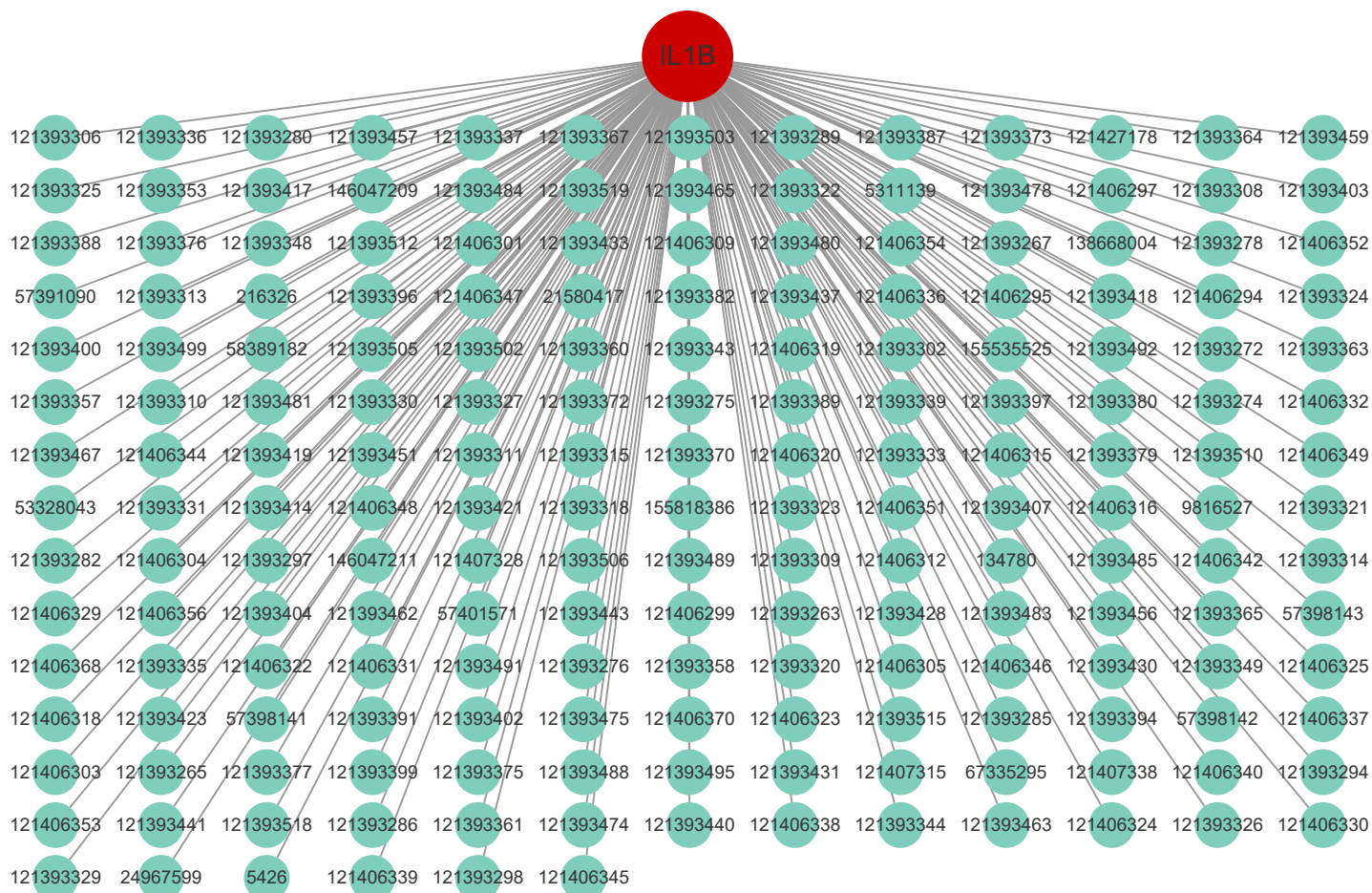

Supplement: Supplementary file 4 [file Data_Sheet_4.PDF]

A

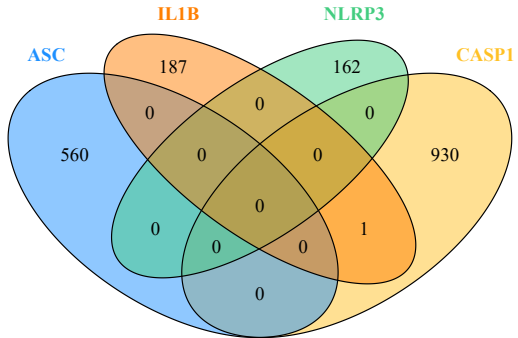

B

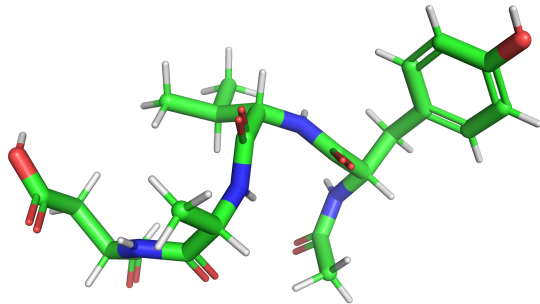

Ac-YVAD-cho

Supplement: Supplementary file 5 [file Data_Sheet_5.PDF]
