# Supplementary material for: Identification of canonical pyroptosis-related genes, associated regulation axis, and related traditional Chinese medicine in spinal cord injury
Source: Front Aging Neurosci. 2023 May 18;15:1152297. doi: 10.3389/fnagi.2023.1152297 (PMC10232751; doi:10.3389/fnagi.2023.1152297)
Supplement: Supplementary file 2 [file Data_Sheet_2.PDF]

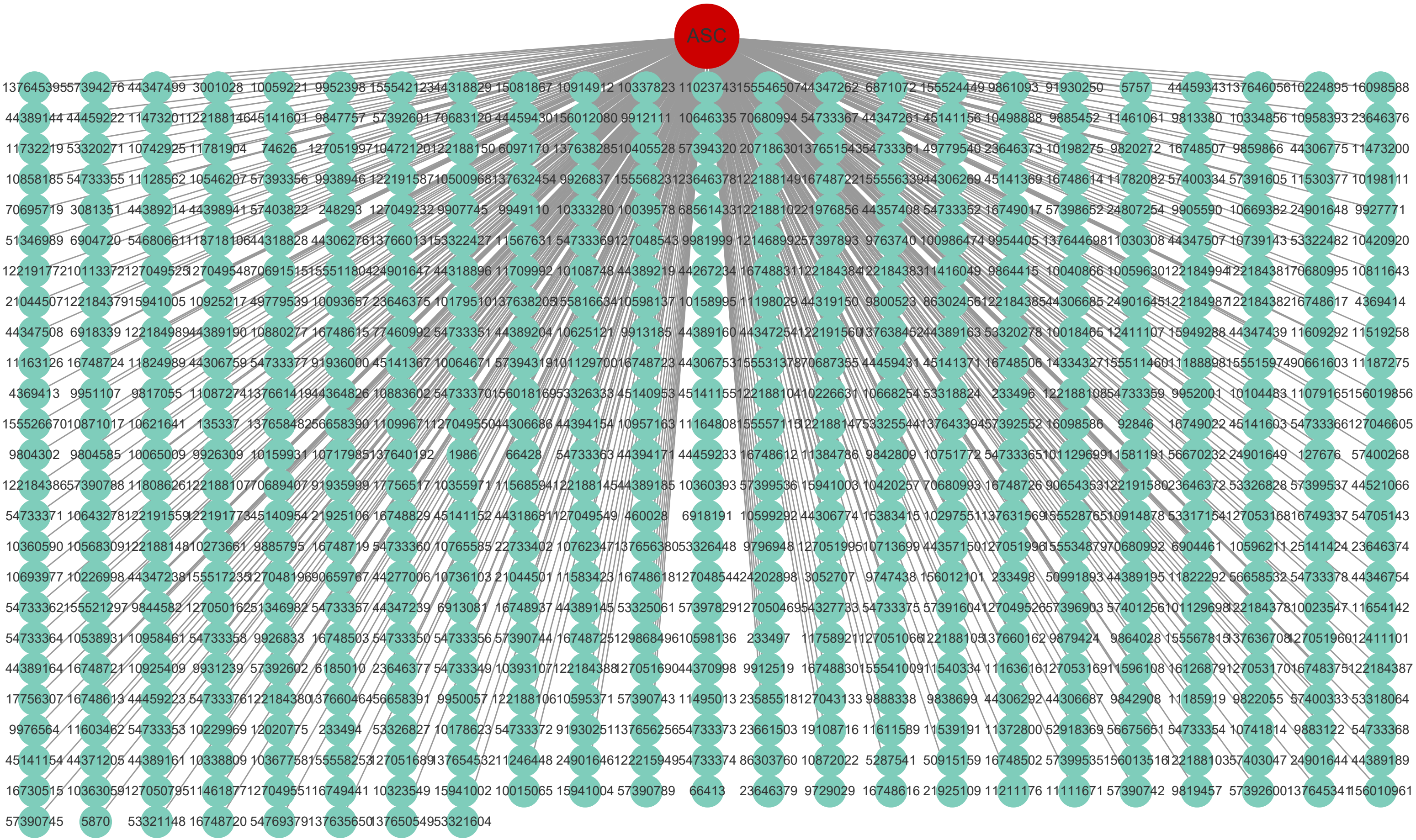
ASC
13764539557394276 44347499 3001028 10059221 9952398 15554212344318829 15081867 10914912 10337823 11023743155546507 44347262 6871072 155524449 9861093 91930250 5757 444593431376460561 0224895 16098588
44389144 44459222 1147320112218814645141601 9847757 57392601 70683120 444594301560120 80 9912111 10646335 70680994 54733367 44347261 45141156 10498888 9885452 11461061 9813380 10334856 10958393 23646376
11732219 53320271 10742925 11781904 74626 127051997104721201221881506097170 13763828510405528 57394320 207186301376515435473336149779540 23646373 10198275 9820272 16748507 9859866 44306775 11473200
10858185 54733355 11128562 10546207 57393356 9938946 122191587105009681376324549926837 1555682312364637812218814916748722155556339443062694514136916748614 11782082 57400334 57391605 11530377 10198111
70695719 3081351 44389214 44398941 57403822 248293 127049232 9907745 9949110 10333280 10039578 685614331221881022197685644357408 54733352 16749017 57398652 24807254 9905590 10669382 24901648 9927771
51346989 6904720 54680661118718106443188284430627613766013153322427 11567631 547333691270485439981999 121468992 57397893 9763740 100986474 9954405 13764469811030308 44347507 10739143 53322482 10420920
1221917721011337212704952512704954870691515155511804249016474431889611709992 10108748 44389219 44267234 16748831122184384122184383114160499864415 10040866 10059630122184994122184381706809951081 1643
2104450712218437915941005 10925217 49779539 10093657 23646375 1017951013763820515581663410598137 10158995 11198029 44319150 9800523 863024561221843854430668524901645122184987122184382167486174369414
44347508 6918339 12218498944389190 10880277 16748615 77460992 54733351 44389204 10625121 9913185 44389160 443472541221915601376384524438916353320278 10018465 12411107 15949288 44347439 11609292 11519258
11163126 16748724 11824989 44306759 54733377 91936000 45141367 10064671 573943191011297001674872344306753155531378706873554445943145141371 16748506 143343271555114601118889815551597490661603 11187275
4369413 9951107 9817055 110872741376614194436482610883602 54733370156018169533263334514095345141155122188104102266311066825453318824 233496 1221881085473335999520011010448311079165156019856
155526670108710171062164113533713765848256658390110996711270495504430668644394154 10957163 1116480815555711512218814753325544137643394573925521609858692846 16749022 45141603 54733366127046605
9804302 9804585 10065009 9926309 10159931 107179851376401921986 66428 54733363 44394171 44459233 16748612 11384786 9842809 10751772 54733365101129699115811915667023224901649 127676 57400268
12218438657390788 1180862612218810770689407 91935999 17756517 10355971 115685941221881454438918510360393 57399536 15941003 10420257 70680993 16748726 906543531221915802364637253326828 57399537 44521066
54733371 106432781221915591221917734514095421925106 16748829 45141152 4431868112704954946002869181911059929244306774 15383415 10297551137631569155528765109148785331715412705316816749337 54705143
10360590 105683091221881481027366198857951674871954733360 10765585 22733402 10762347137656380533264489796948 12705199510713699443571501270519961555348797068099269044611059621125141424 23646374
10693977 10226998 443472381555172351270481969065976744277006 10736103 21044501 11583423 1674861812704854424202898 3052707 9747438 156012101 233498 50991893 44389195 11822292 56658532 54733378 44346754
547333621555212979844582127050162513469825473335744347239 6913081 16748937 44389145 53325061 573978291270504695432773354733375 573916041270495265739690357401256101129698122184378100235471165 4142
54733364 10538931 10958461 54733358 9926833 16748503 54733350 54733356 57390744 16748725129868496105981362334971175892112705106812218810513766016298794249864028155567815137636708127051960124 11101
44389164 16748721 10925409 9931239 57392602 6185010 23646377 54733349 10393107122184388127051690443709989912519 167488301555410091154033411163616127053169115961081612687912705317016748375122184387
17756307 16748613 44459223 54733376122184380137660464566583919950057 1221881061059537157390743 11495013 23585518127043133 9888338 9838699 44306292 44306687 9842908 11185919 9822055 57400333 53318064
9976564 11603462 54733353 10229969 12020775 233494 53326827 10178623 54733372 91930251137656256547333732366150319108716 11611589 11539191 11372800 52918369 56675651 54733354 10741814 9883122 54733368
45141154 44371205 44389161 10338809 10367758155558253127051689137654532112464482490164612221594954733374 86303760 10872022 5287541 50915159 16748502 573995351560135161221881035740304724901644 44389189
16730515 1036305912705079511461877127049551167494411032354915941002 10015065 15941004 57390789 66413 23646379 9729029 16748616 21925109 11211176 11111671 57390742 9819457 57392600137645341156010961
57390745 5870 53321148 16748720 547693791376356501376505495332 1604
