# Supplementary material for: Identification of canonical pyroptosis-related genes, associated regulation axis, and related traditional Chinese medicine in spinal cord injury
Source: Front Aging Neurosci. 2023 May 18;15:1152297. doi: 10.3389/fnagi.2023.1152297 (PMC10232751; doi:10.3389/fnagi.2023.1152297)

A

Homo sapiens	Mus musculus	Rattus norvegicus
GSDMA	Gsdma3/Gsdma2/Gsdma	Gsdma
GSDMB	——	——
GSDMC	Gsdmc/Gsdmc2/Gsdmc3/Gsdmc4	Gsdmc
GSDMD	Gsdmd	Gsdmd
GSDME	Gsdme	Gsdme

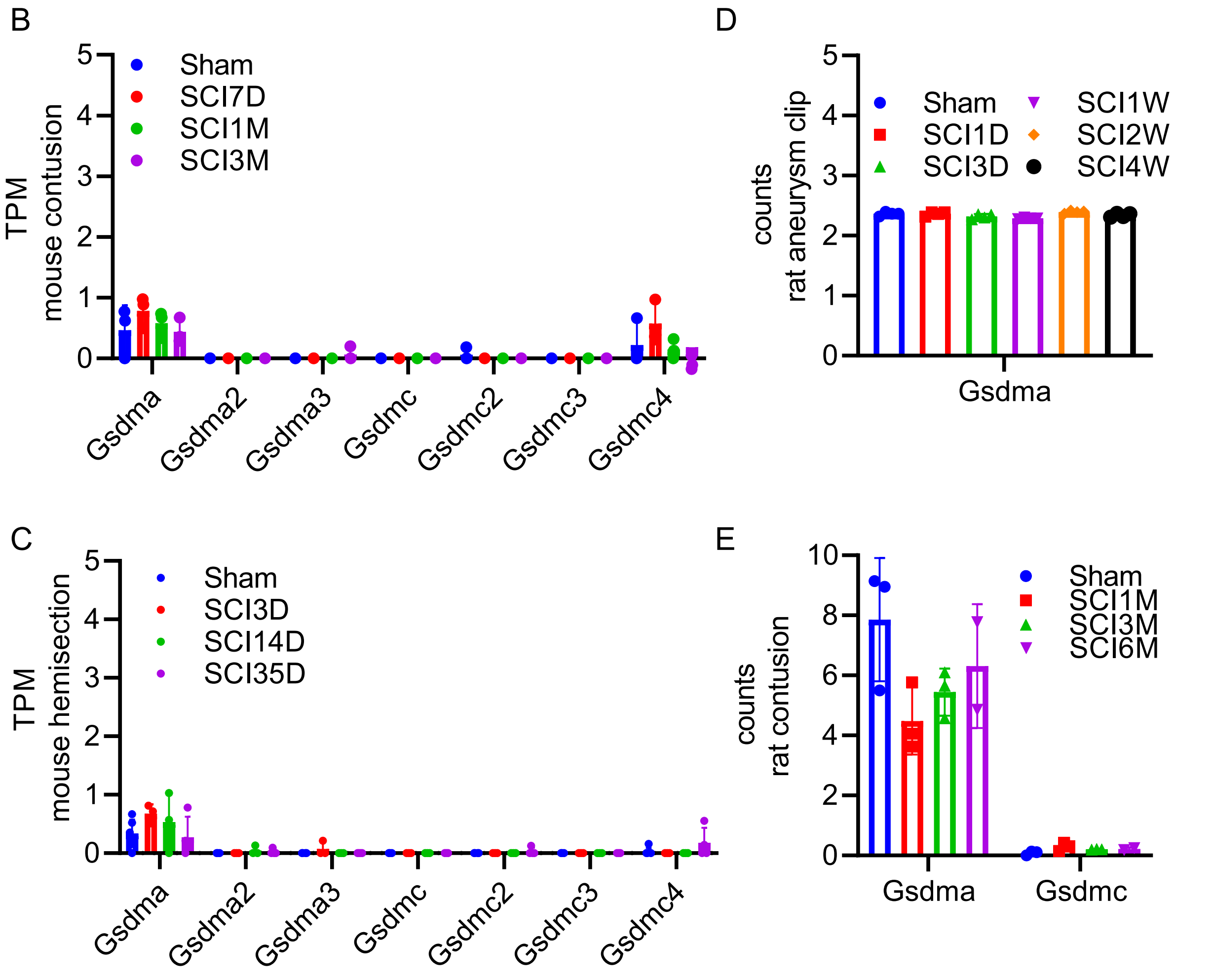

Supplement: Supplementary file 6 [file Data_Sheet_6.PDF]
