# Supplementary material for: Identification of canonical pyroptosis-related genes, associated regulation axis, and related traditional Chinese medicine in spinal cord injury
Source: Front Aging Neurosci. 2023 May 18;15:1152297. doi: 10.3389/fnagi.2023.1152297 (PMC10232751; doi:10.3389/fnagi.2023.1152297)
Supplement: Supplementary file 7 [file Table_1.DOCX]

**Figure S1** Small molecular (PubChem CID)- NLRP3 network

**Figure S2** Small molecular (PubChem CID)- ASC network

**Figure S3** Small molecular (PubChem CID)-CASP1 network

**Figure S4** Small molecular (PubChem CID)- IL1B network

**Figure S5** **(A)**Venn plot of small moleculars in small molecular- NLRP3/PRGs network **(B)** 3D Structure of Ac-Yvad-cho

**Figure S6** **(A)**Gsdmin family members **(B)** the expression of Gsdmin family members in mouse hemisection model **(C)** the expression of Gsdmin family members in mouse moderate contusive model **(D)** the expression of Gsdmin family members in rat aneurysm clip impact-compression model **(E)** the expression of Gsdmin family members in rat moderate contusive model
